# Apoptotic and Early Innate Immune Responses to PB1-F2 Protein of Influenza A Viruses Belonging to Different Subtypes in Human Lung Epithelial A549 Cells

**DOI:** 10.1155/2018/5057184

**Published:** 2018-12-31

**Authors:** Gunisha Pasricha, Sanjay Mukherjee, Alok K. Chakrabarti

**Affiliations:** Microbial Containment Complex, National Institute of Virology, Sus Road, Pashan, Pune 411021, India

## Abstract

PB1-F2 is a multifunctional protein and contributes to the pathogenicity of influenza A viruses. PB1-F2 is known to have strain and cell specific functions. In this study we have investigated the apoptotic and inflammatory responses of PB1-F2 protein from influenza viruses of diverse pathogenicities in A549 lung epithelial cells. Overexpression of PB1-F2 resulted in apoptosis and heightened inflammatory response in A549 cells. Comparison revealed that the response varied with each subtype. PB1-F2 protein from highly pathogenic H5N1 virus induced least apoptosis but maximum inflammatory response. Results indicated that apoptosis was mediated through death receptor ligands TNF*α* and TRAIL via Caspase 8 activation. Significant induction of cytokines/chemokines CXCL10, CCL5, CCL2, IFN*α*, and IL-6 was noted in A549 cells transfected with PB1-F2 gene construct of 2008 West Bengal H5N1 virus (H5N1-WB). On the contrary, PB1-F2 construct from 2007 highly pathogenic H5N1 isolate (H5N1-M) with truncated N-terminal region did not evoke as exuberant inflammatory response as the other H5N1-WB with full length PB1-F2, signifying the importance of N-terminal region of PB1-F2. Sequence analysis revealed that PB1-F2 proteins derived from different influenza viruses varied at multiple amino acid positions. The secondary structure prediction showed each of the PB1-F2 proteins had distinct helix-loop-helix structure. Thus, our data substantiate the notion that the contribution of PB1-F2 to influenza pathogenicity is greatly strain specific and involves multiple host factors. This data demonstrates that PB1-F2 protein of influenza A virus, when expressed independently is minimally apoptotic and strongly influences the early host response in A549 cells.

## 1. Introduction

Influenza is pathogenic viral disease, causing the emergence of newer epidemics and pandemics in mammals [1, 2]. Since the beginning of 20th century, there have been four pandemics: Spanish influenza (H1N1) in 1918/1919, Asian influenza (H2N2) in 1957, Hong Kong influenza (H3N2) in 1968, and H1N1 influenza in 2009 [3, 4]. Influenza A virus (IAV) causes acute respiratory inflammation in humans and symptoms include high fever, body aches, and fatigue [4]. Symptoms of IAV infection is mostly mild in humans but may progress to fatal viral pneumonia if the virus spreads from the upper airways to the alveolar space in the lower respiratory tract [4].

IAV belongs to the Orthomyxoviridae family and its genome consists of eight negative strand RNA segments [4, 5]. PB1-F2 was the first accessory protein discovered in IAV [6]. Subsequently, more proteins like PB1-N40 and PA-N155 have been discovered [4, 5]. It is important to study these novel proteins in order to evaluate their role in the pathogenesis of IAV infection. PB1-F2 is the second protein encoded by the +1 alternate open reading frame within the PB1 gene. It is translated from the fourth initiation codon via a leaky ribosomal scanning mechanism wherein 43S ribosomal complex bypasses the PB1 start codon and two additional intervening AUG codons [6, 7]. PB1-N40 is the third protein translated from the fifth initiation codon of PB1 gene. Proteins encoded by PB1 gene have translational interdependence, which makes it difficult to understand their relative contributions to the replication cycle and pathogenicity of IAV [4, 5].

PB1-F2 has been shown to have proapoptotic activity when expressed either independently or during influenza virus infection [6]. The C-terminal region of PB1-F2 interacts with mitochondrial antiviral signaling protein (MAVS), resulting in decreased mitochondrial membrane potential and ultimately ensuing in apoptosis [8]. PB1-F2 has also been implicated in regulation of polymerase activity, exacerbating pathogenicity in animal models, causing susceptibility to secondary bacterial infection and regulation of innate immune response [9].

PB1-F2 has been discovered more than a decade ago and yet there is not much clarity about its pathogenicity determinants. The major deterrent in this is the fact that PB1-F2 protein is highly variable in many of the subtypes of IAV and truncated either from the C-terminal or N-terminal end, thus questioning its evolutionary utility in different subtypes and hosts [10]. Thus, it is not surprising that the effects of the PB1-F2 are strain, cell type, and host specific [11, 12]. Also, much of the research on PB1-F2 has been done using only a few important viral isolates, such as A/Puerto Rico/8/34 (PR8), A/WSN/1933 (WSN), 1918 Spanish flu belonging to H1N1 subtype and avian influenza A H5N1 isolates [13–16]. Thus, it is necessary to study and compare PB1-F2 protein from IAV isolates from different host species and have a complete understanding of how PB1-F2 proteins contribute to IAV replication or virulence.

In this study, a deeper understanding of PB1-F2 protein from various IAVs and their role in modulation of host cell response was sought. We used PB1-F2 gene of five different IAVs, belonging to four avian and one human isolate. Two isolates of highly pathogenic H5N1 viruses having PB1-F2 protein of different sizes were included in this study. The H5N1-M has an N-terminal truncation in PB1-F2 sequence and does not produce a complete PB1-F2 protein [17] while H5N1-WB produces a full length PB1-F2 protein [18]. We attempted to study the effects of PB1-F2 expression and understand its ability to initiate apoptosis and subvert or subdue the host immune response in A549 cells. We studied the PB1-F2 induced host responses in human lung epithelia since respiratory tract is the entry portals of influenza virus and plays a key role in the initial host response [19]. Most of* in vitro* studies on PB1-F2 have been carried out in monocyte/macrophage cell lines. Therefore, it seemed important to study the role of PB1-F2 in inducing apoptotic responses and cytokine production in epithelial cells of the respiratory mucosa.

## 2. Material and Methods

### 2.1. Sequence Analysis and Secondary Structure Prediction of PB1-F2 Proteins

PB1-F2 sequences were aligned and analyzed using ClustalX2 version 2.1 program. Secondary structure prediction of the PB1-F2 was performed using RaptorX program (http://raptorx.uchicago.edu/) [20].

### 2.2. Cell Line, Viruses and Reagents

Madin-Darby Canine Kidney (MDCK) and human lung epithelial (A549) cell lines were obtained from ATCC and were maintained in Dulbecco's modified Eagle's medium (DMEM; Gibco, San Diego, CA) with 10% fetal bovine serum (Gibco, San Diego, CA) in tissue culture flasks (Corning, USA) at 37°C in a CO_2_ incubator. Plasmid constructs of PB1-F2 gene were made by amplifying a short region of PB1 gene from five strains of IAVs belonging to different subtypes. These included four avian influenza viruses A/chicken/India/WBNIV2653/2008 (H5N1-WB); A/chicken/India/NIV9743/2007 (H5N1-M); A/chicken/India/WB-NIV1057231/2010 (H9N2); and A/aquaticbird/India/NIV-17095/2007 (H11N1). PB1-F2 of human influenza A H1N1 (A/WSN/33) was amplified from a plasmid construct of PB1.

To test the transfection efficiency of the A549 cell line, cells were transfected with pmax- GFP vector (Lonza Group, Switzerland) and the fluorescence was detected using GFP antibody (FL), a rabbit polyclonal IgG (SantaCruz Biotechnology, CA). The other antibodies obtained from Santa Cruz Biotechnology, CA, USA, were *β*-Actin mouse monoclonal antibody (ACTBD11B7), goat anti-mouse IgG-Horse Radish Peroxidase (HRP), and mouse anti-rabbit IgG-HRP. Anti-V5 and anti-His (C-term) mouse monoclonal antibodies (Life technologies, CA, USA) were used to detect expression of PB1-F2 fusion protein from pcDNA™3.1/V5-His-TOPO plasmid (Life technologies, CA, USA). To detect the expression of PB1-F2 protein, at 8 h post transfection, proteasome inhibitor MG132 and calpain inhibitor were added alone and in combination at concentration of 10*μ*M and 20*μ*M respectively into the cell line.

### 2.3. RNA Extraction and RT-PCR

The viruses were grown and propagated in MDCK cells. The cell culture supernatant was used to extract viral RNA with QIAamp Viral RNA Mini Kit (Qiagen, Germany). Reverse transcription was carried out using Superscript II RT-PCR kit (Invitrogen, Carlsbad, CA, USA) and Uni12 primer [21]. PB1-F2 genes were amplified from the above-mentioned viral strains using AccuPrime* Taq* DNA polymerase system (Invitrogen, Carlsbad, CA, USA). The subtype specific primers which were used for the amplification are mentioned in the Table 1. The PCR program for all the amplifications was as follows: initial denaturation at 94°C for 2 mins followed by 30 cycles of 94°C for 30 secs, 55°C for 30 secs, and 68°C for 1 min. The final product was stored at 4°C.

### 2.4. Expression Plasmid Construction and Transient Transfection

Amplified products of the PB1-F2 gene from all the five cDNA were cloned into pcDNA 3.1/V5-His-TOPO expression vector (Invitrogen, Carlsbad, CA, USA). Competent* E. coli* DH5*α* cells were transformed with these constructs. The constructs containing PB1-F2 insert were isolated and purified using plasmid Midi kit (Qiagen, Germany). All these constructs were confirmed by restriction digestion and further reconfirmed by sequencing.

Plasmid constructs containing PB1-F2 gene from the different subtypes were transfected in A549 cells using Lipofectamine 2000 (Invitrogen, Carlsbad, CA, USA) according to the manufacturer's protocol. Transfection mixtures were then gently added to the respective wells. Twenty-four hours after transfection, total RNA and protein were extracted. The transfection efficiency of A549 cells was determined by transfecting the cell line with pmax-GFP vector (Lonza Group, Switzerland). Untransfected cells served as cell control and cells transfected with the empty expression vector pcDNA3.1 served as mock control.

### 2.5. PB1-F2 mRNA and Protein Expression in Transfected Cells

Twenty-four hours post transfection cells were harvested and RNA was extracted using TRIzol® Reagent (Invitrogen, Carlsbad, CA, USA). RNA was purified using RNeasy mini kit (Qiagen, Carlsbad, CA) and their yields were evaluated in a Nanodrop spectrophotometer (Nanodrop technologies, Wilmington, DE, USA) at 260 nm. To determine the mRNA expression of PB1-F2 gene in A549 cells, 500 ng of RNA was reverse transcribed using the Superscript II RT-PCR kit (Invitrogen, Carlsbad, CA, USA) and Uni12 primer and the PB1-F2 gene was amplified as mentioned above.

The total cellular protein was extracted using radio-immunoprecipitation assay (RIPA) buffer (Sigma, St. Louis, Missouri, USA). Protease inhibitor and Phosphatase inhibitor cocktails (Calbiochem, USA) were added to RIPA lysis buffer. After protein extraction the cellular debris was pelleted by centrifugation at 8000*g *for 10 min at 4°C. The protein concentration of the supernatant was determined by Nanodrop spectrophotometer (Nanodrop technologies, Wilmington, DE, USA) at 280 nm. Equal concentration of cell extract was fractioned on 12.5% Sodium dodecyl sulfate-polyacrylamide gel electrophoresis (SDS-PAGE) and the separated proteins were transferred by semi-dry blotting onto nitrocellulose membranes (Hybond C-Extra; GE Healthcare) for reaction with specific antibodies against the His-tagged proteins.

### 2.6. TUNEL Assay

For detection and relative quantification of apoptosis in A549 cells transfected with PB1-F2 construct from various influenza subtypes, the TUNEL Apoptosis Detection assay was performed (terminal deoxynucleotidyl transferase mediated dUTP nick end labeling), using a kit from APO-BrdU™ TUNEL Assay Kit (Invitrogen Life Technologies, Carlsbad, CA, USA). TUNEL assay detects the DNA fragmentation of apoptotic cells by exploiting the 3′-hydroxyl ends of the DNA breaks. These hydroxyl groups are then modified by terminal deoxynucleotidyl transferase (TdT) enzyme, which adds deoxyribonucleotides in a template-independent fashion. Addition of the deoxythymidine analog 5-bromo-2′-deoxyuridine 5′-triphosphate (BrdUTP) to the TdT reaction serves to label the break sites. Once incorporated into the DNA, BrdU can be detected by an anti-BrdU antibody using standard immunohistochemical techniques. The nuclear staining was performed with propidium iodide (PI). The assay was carried out according to the instructions of the manufacturer and slides were visualized using Olympus IX51 microscope. Apoptotic index (AI) was determined. AI = number of TUNEL-positive epithelial cells/total number of epithelial cells stained with PI from a total of 25 fields per sample.

### 2.7. Quantitative Real-Time PCR (qRT-PCR)

Total cellular RNA from control and transfected A549 cells was extracted using TRIzol reagent (Invitrogen Life Technologies, Carlsbad, CA, USA) and purified using RNeasy mini kit (Qiagen, Carlsbad, CA). Comparison of the relative expression of 84 apoptosis-related genes between the samples was made with Human Apoptosis RT^2^ Profiler PCR Array (SABiosciences, Frederick, MD). The total RNA was treated with DNase I (Roche Diagnostic, Germany) to eliminate genomic DNA contamination at 37°C for 20 minutes. One microgram of total RNA was converted to cDNA using RT^2^ First Strand cDNA Synthesis kit (Qiagen, Carlsbad, CA) following the manufacturer's protocol. The cDNA was used for qPCR amplification in the Human Apoptosis RT^2^ Profiler PCR Array (SABiosciences, Frederick, MD) using an ABI Prism 7300 detection system (Applied Biosystems, Foster City, CA) according to manufacturer's instructions. The PCR conditions and cycles were as follows: initial DNA denaturation 10 min at 95°C, followed by 40 cycles at 95°C for 15 sec, and final step at 60°C for 1 min. Melting curves were analyzed to determine the specificity of each reaction to the target. Reactions were conducted in duplicate and repeated at least three times for each experiment, and the mean values and standard deviations were calculated. To ensure that the primers produced a single and specific PCR amplification product, a dissociation curve was performed at the end of the PCR cycle. The data were presented as the fold change using the formula 2^-ΔΔCT^ as recommended by the manufacturer.

### 2.8. ELISA Based Cytokine Quantification in Cell Supernatants

Cytokine levels (IL-1*β*, IL-6, IL-8, TNF-*α*, CCL2, CCL-5, CXCL10, and CXCL9) in supernatants of control and transfected cells were analyzed using human viral induced cytokines Multi-Analyte ELISArray kit (SABiosciences, Frederick, MD). Supernatants (1:2 diluted) were incubated in 96-well plates precoated with individual cytokine capture antibodies for 2 hours. After they were washed, biotin-conjugated cytokine detection antibodies were added for 30 minutes. Bound antibodies were detected with avidin-HRP and development solution. Color reactions were quantified at OD 450. Cytokine concentrations were determined by extrapolation from cytokine standard curves, according to the manufacturer's protocol. The experiments were repeated three times using supernatant from cells transfected with individual PB1-F2 constructs.

### 2.9. Statistical Analysis

Statistical analysis was carried out using GraphPad Prism 5.0 software (San Diego, CA, USA) by applying the Student t-test for 2 group comparisons. All the experiments were repeated at least 3 times for validation. The differences were considered significant at p < 0.05.

## 3. Results

In this study, we used PB1-F2 protein belonging to different subtypes of IAV and investigated its ability to induce apoptosis and modulate inflammatory response in transfected A549 cells.

### 3.1. Amino Acid Sequence Analysis and Secondary Structure Prediction of PB1-F2 Protein from Different Influenza A Viruses

**Figure 1:**
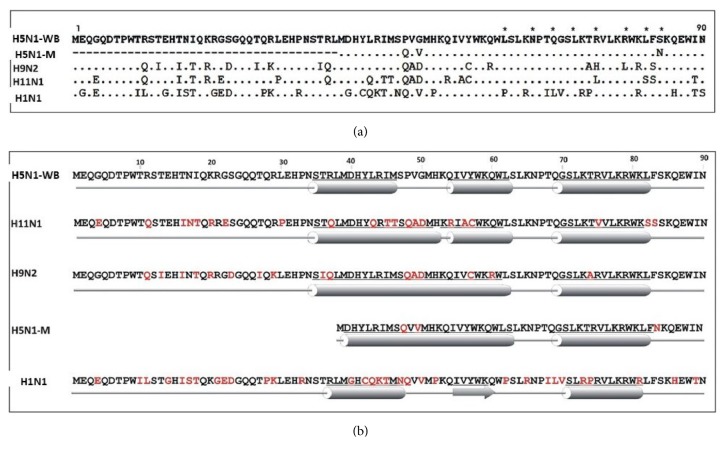
PB1-F2 sequence comparison and structural predictions. (a) Amino acid multialignment of the five PB1-F2 variants included in this study. PB1-F2 proteins analyzed* in silico *in this study from the influenza viruses are abbreviated as follows: A/chicken/India/WBNIV2653/2008 (H5N1-WB); A/chicken/India/NIV9743/2007 (H5N1-M); A/chicken/India/WB-NIV1057231/2010 (H9N2); A/aquatic bird/India/NIV-17095/2007 (H11N1); and A/WSN/1933 (H1N1). Eight amino acid residues which have been reported in literature to have significance in enhancing apoptosis and inflammation have been marked with asterisk. L62, R75, R79, and L82 are reported as inflammatory motifs, N66S as a pathogenic marker, L69 and L75 important for mitochondrial localization, K73 and R75 minimally required for apoptosis via mitochondria. (b) Secondary structure predictions were obtained with the software RaptorX program. Straight line structure represents the loop, cylinder represents the *α* Helix, and arrow head marks the *β* sheet structure.

The length and sequence of PB1-F2 protein greatly decide its contribution to viral pathogenicity. We compared the amino acid sequence of the PB1-F2 protein of the five IAVs, four of them were isolated from avian species while one of them from mammals. Since evolutionarily IAV moved from avian hosts to mammals [22], the amino acid sequence was compared with PB1-F2 protein of highly pathogenic avian influenza-H5N1 (H5N1-WB) virus. The amino acid alignment of PB1-F2 sequences showed variations at more than half of the amino acids positions (52/90), 19 of which were contributed by H1N1 (A/WSN/1933) alone. Avian influenza viruses in the study varied at 26 amino acid positions, hence showing 71.1% homology. All these variations were distributed in the entire length of the protein (Figure 1(a)).

Amino acid residues which are reported to be involved in modulating apoptosis and host immune responses are depicted in Figure 1(a). The PB1-F2 proteins from H5N1-WB and H5N1-M have amino acids residues L62, R75, R79, and L82 which are inflammatory motifs of the protein [23]. Except for mammalian H1N1, PB1-F2 protein from all other subtypes isolated from avian species had Leucine at amino acid position 62. Double Leucine residue at positions 69 and 75 which is required for mitochondrial localization of PB1-F2 was not present in any of the strains. Amino acids K73 and R75 which are minimally required for apoptosis via mitochondria were present in the PB1-F2 sequence of both the H5N1-viruses. All the viruses had a full length 90 amino acid PB1-F2 protein except for H5N1-M which had an N-terminal truncation and was only 52 amino acid long.

The secondary structures of all the PB1-F2 proteins showed a typical helix-loop-helix structure except for H1N1 (WSN) which showed a small *β* sheet structure in between *α*-helices (Figure 1(b)). PB1-F2 structure of avian influenza viruses varied from each other in the length of their alpha-helices. Distinctive two *α*-helix breaks were seen for viruses isolated from avian species with a single break for H9N2 and H5N1-M but double breaks for H5N1-WB and H11N1 viruses. The *α*-helices towards the C-terminal end of PB1-F2 have been reported to be associated with viral pathogenicity [22]. Interestingly, PB1-F2 of H1N1-WSN displayed a unique *β*-sheet structure (54-59aa) in between the two *α* helices (Figure 1(b)).

### 3.2. Apoptosis in A549 Cells Transfected with PB1-F2 constructs of Different Influenza A Viruses

**Figure 2:**
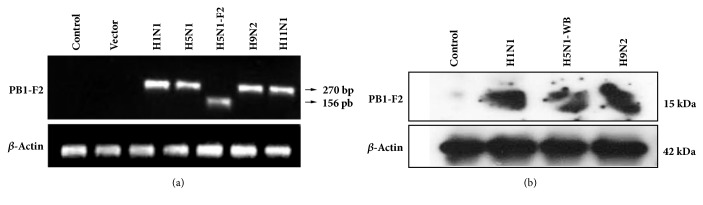
A549 cells grown in a 60 mm culture dish and transfected with different PB1-F2 constructs and empty pcDNA3.1 plasmid as vector (mock) control. Forty-eight-hour after transfection, the cells were lysed and PB1-F2 expression was detected by (a) reverse transcriptase PCR and (b) immunoblotting using a monoclonal mouse anti-HIS antibody. PB1-F2 protein expression analyzed in this study is abbreviated as follows: A/chicken/India/WBNIV2653/2008(H5N1-WB); A/chicken/India/NIV9743/2007 (H5N1-M); A/chicken/India/WB-NIV1057231/2010 (H9N2); A/aquatic bird/India/NIV-17095/2007 (H11N1); and A/WSN/1933 (H1N1).

We expressed PB1-F2 protein of different influenza A viruses in A549 cells and investigated its ability to induce apoptosis in host cells using TUNEL assay and by studying the expression of apoptotic genes. The expression of PB1-F2 was confirmed at mRNA and protein level as shown in Figure 2. We could not able to detect expression of PB1-F2 protein in H5N1-M and H11N1 viruses in western blots. This could be due to the small size and shorter half-life of the protein in these subtypes (Figure 2(b)).

**Figure 3:**
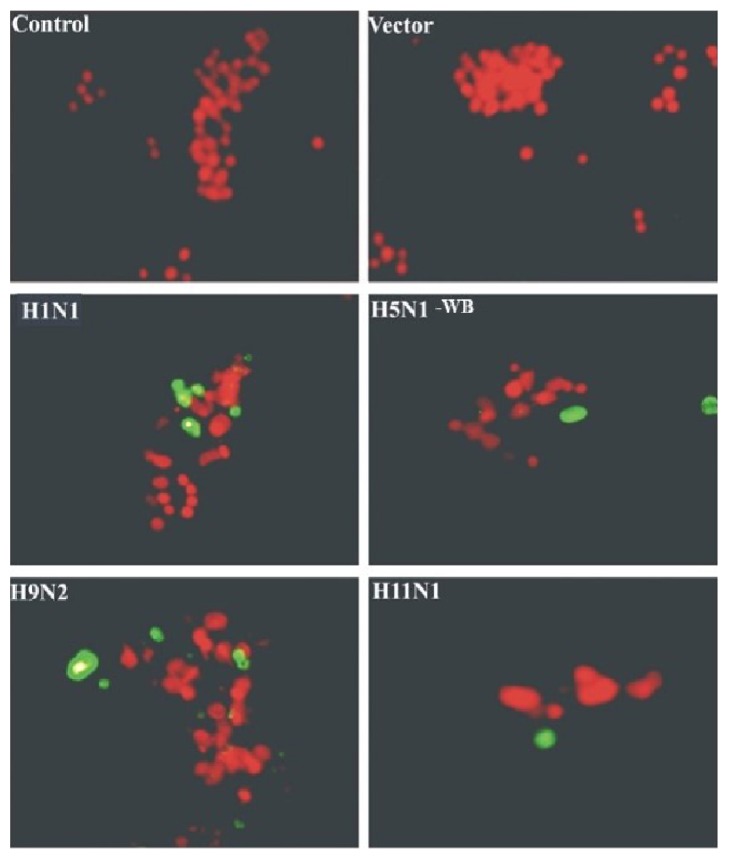
Ectopic expression of PB1-F2 protein predisposes cells to minimal apoptosis. A549 cells were transfected with PB1-F2 constructs and the apoptosis was observed by TUNEL staining with FITC-conjugated dUTP cells showing apple green fluorescence which were apoptotic. The overall proportion of death cells was measured by propidium iodide staining (seen as red in color).

TUNEL assay revealed that only a small proportion of PB1-F2 transfected cells underwent apoptosis (Figure 3). This could explain the relatively low proapoptotic effect of the PB1-F2 protein observed in the assay above. Number of apoptotic cells was counted from a population of more than 500 cells. At 24 h post-transfection, highest proportion (20%) of apoptotic cells were observed with H1N1 (WSN) PB1-F2 construct compared to 15% with H11N1-PB1-F2 and least with H5N1 (WB) PB1-F2 (5%).

To further explore the molecular mechanism of apoptosis triggered by transient expression of PB1-F2 protein belonging to various influenza subtypes in A549 cell line, we determined the expression of 84 key genes involved in programmed cell death by using the Human Apoptosis RT^2^ Profiler PCR Array. This array includes TNF ligands and their receptors, members of the bcl-2, caspase, IAP, TRAF, CARD, death domain, death effector domain, and CIDE families, as well as genes involved in the p53 and DNA damage pathways.

The mRNA expression profile showed that 24 of the 84 genes analyzed in this study were upregulated with statistical significance (p<0.05) when compared with the mock control (empty vector). We grouped the gene transcripts in 4 groups based on function and signaling pathway. Group I included genes belonging to the TNF death receptors and ligand family, Group II included different caspases, Group III belonged to BCl-2 family member genes while Group IV belonged to different mitochondrial cell death inhibitor (IAP) genes involved in intrinsic regulation of apoptosis.

**Figure 4:**
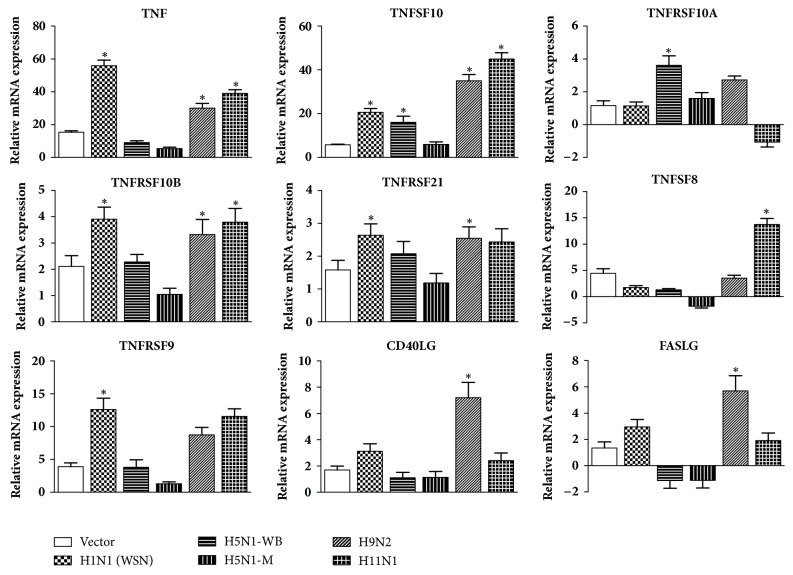
Impact of PB1-F2 expression on the mRNA expression levels of death receptor ligands and receptors. Gene expressions were normalized with the *β*-actin gene expression level and presented as fold increase relative to nontransfected cell controls. Data asterisks (*∗*) indicate (*p∗* < 0.05) and are calculated relative to the mock vector control which is also represented in the figure. This data is representative of three independent experiments. Error bars represent the ± SEM.

Analysis of group I genes revealed that there was statistically significant upregulation of expression of* TNFα* in A549 cells in response to PB1-F2 protein from H1N1 (WSN), H9N2, and H11N1 when compared to mock control. Most of the TNF family member genes showed significant higher expression (compared to mock) in response to PB1-F2 protein belonging to low pathogenic avian influenza viruses and WSN strain. The expression level of* TNF, TNFRSF9, TNFRSF10a,* and* TNFRSF21* was highest in H1N1-WSN of all the subtypes used for the study. However, expression of* TNFSF10* and* TNFRSF10a* genes was significantly higher in cells transfected with PB1-F2 of highly pathogenic avian influenza-H5N1-WB virus (Figure 4).

**Figure 5:**
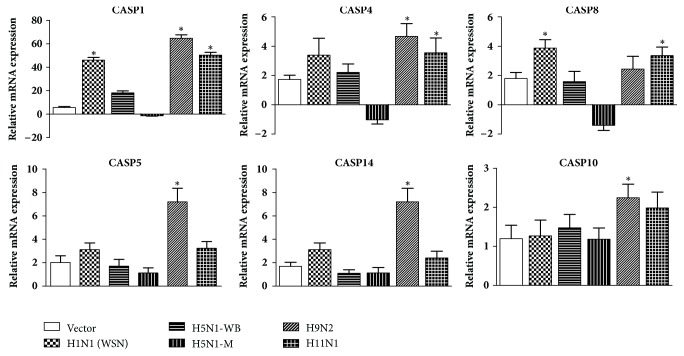
Impact of PB1-F2 expression of the mRNA expression levels of Caspases. Gene expressions were normalized with the *β*-actin gene expression level and presented as fold increase relative to nontransfected cell controls. Asterisks (*∗*) indicate (*p∗* < 0.05) and are calculated relative to the mock vector control which is also represented in the figure. This data is representative of three independent experiments. Error bars represent the ± SEM.

Analysis of gene expression of caspase family (Group II) revealed that Caspase 8 (*CASP8*) was significantly upregulated in response to PB1-F2 of influenza A H1N1 and H11N1 strains. Caspase 10 (*CASP10*) and Caspase 14 (*CASP14*) which are in turn activated by* CASP8* were significantly upregulated in response to PB1-F2 of H9N2 strain (Figure 5). The gene expression of Caspase 1 was found considerably higher (>10 folds) in response to PB1-F2 of H1N1, H9N2, and H11N1 subtypes (Figure 5). Overall,* CASP1, CASP4, CASP5, *and* CASP14* showed highest expression in response to PB1-F2 protein belonging to H9N2 subtype.

**Figure 6:**
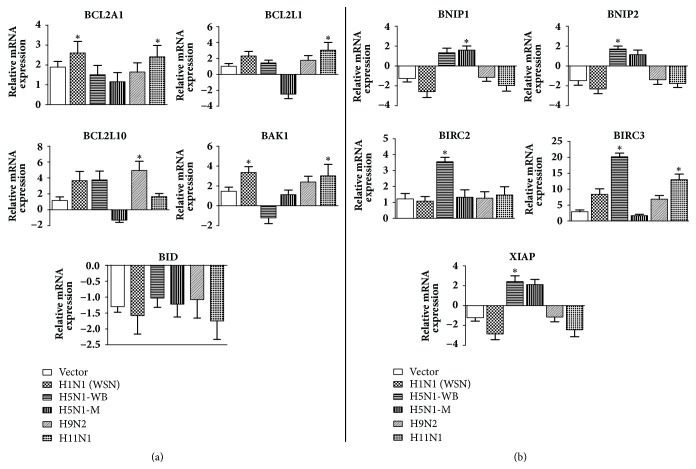
Impact of PB1-F2 expression on the mRNA expression levels of (a) BCl2 member family which govern the permeabilization of mitochondrial outer membrane. (b) IAP (Inhibitors of Apoptosis) and other antiapoptotic proteins. Gene expressions were normalized with the *β*-actin gene expression level and presented as fold increase relative to nontransfected cell controls. Data Asterisks (*∗*) indicate (*p∗* < 0.05) and are calculated relative to the mock vector control which is also represented in the figure. This data is representative of three independent experiments. Error bars represent the ± SEM.

Group III include genes belong to the BCL2 gene family which are both pro- and anti-apoptotic. BAK1 expression was significantly higher in response to PB1-F2 protein of H1N1 and H11N1 viruses (Figure 6(a)). The BCL2 ligands,* BCL2L1 *and* BCL2L10,* showed highest expression in H11N1 and H9N2, respectively.* BID* was downregulated in response to PB1-F2 of all the subtypes, although it was found to be not statistically significant (Figure 6(a)).

Group IV genes included IAPs (inhibitors of apoptotic protein: BIRC2 and BIRC3) and apoptosis protectors (BNIP1 and BNIP2) (Figure 6(b)). The gene expression of IAPs showed significant upregulation in A549 cells transfected with PB1-F2 constructs of H5N1. Interestingly,* BNIP1, BNIP2, *and* XIAP* genes were downregulated by PB1-F2 of all subtypes except for H5N1 (Figure 6(b)).

### 3.3. Cytokine Expression in A549 Cells Transfected with PB1-F2 Constructs of Different Influenza A Viruses 

**Figure 7:**
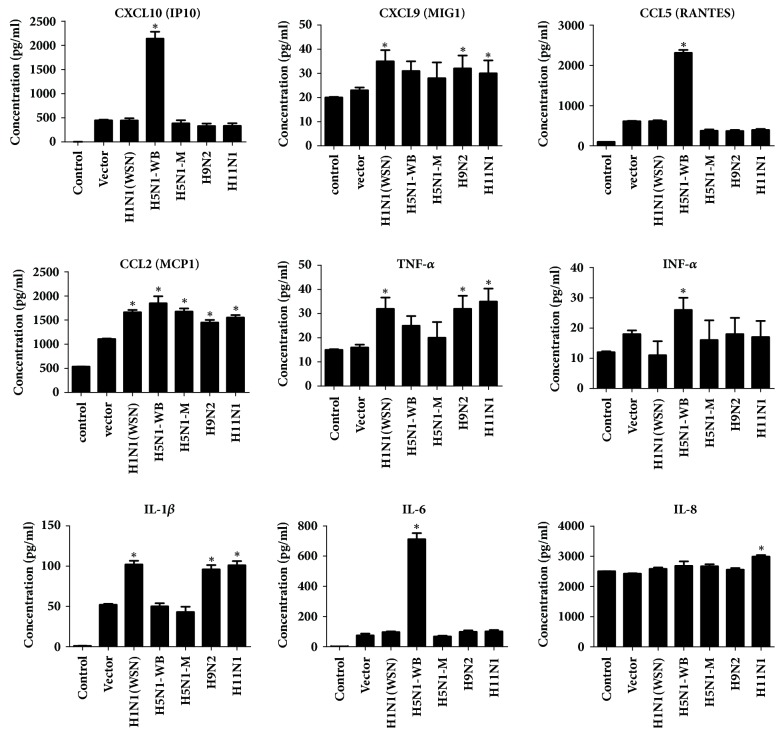
Levels of secreted cytokine/chemokine levels in response to PB1-F2 expression in A549 cells measured by ELISArrays. Data are presented as absorbance values at 450 nm. Concentration of the cytokine/chemokines is in pg/ml. Asterisks (*∗*) indicate (*p∗* < 0.05) and are calculated relative to the mock vector control which is also represented in the figure. This data is representative of three independent experiments. Error bars represent the ± SEM.

To understand and compare the proinflammatory effect of PB1-F2 protein from various influenza subtypes in A549 cell, we measured the expression of cytokines/chemokines in supernatants of PB1-F2 transfected cells using Multi-Analyte ELISArray Kit (Figure 7). The expression levels of inflammatory cytokines INF*α*, IL-6, MCP1 (CCL2), RANTES (CCL5), and IP-10 (CXCL10) were considerably and significantly (p<0.05) higher in H5N1-WB PB1-F2 transfected cells compared to mock control (empty vector) at 24 h post transfection. The levels of TNF*α* were significantly higher (p<0.05) for PB1-F2 protein from H1N1 (WSN), H9N2, and H11N1 viruses and this was in concurrence with the gene expression data. IFN*α* levels induced by H1N1 (WSN) PB1-F2 were lower than the level in control cells although it was not statistically significant. On the contrary the IFN*α* levels were significantly higher (p<0.05) in cells transfected with H5N1-WB PB1-F2 construct. Overall, highly pathogenic avian influenza H5N1-WB subtype was the strongest inducer for most cytokines/chemokines showing prominent induction for CXCL10, RANTES, INF-*α*, and IL-6 in transfected cells (Figure 7).

## 4. Discussion

PB1-F2 is a nonstructural viral protein and has been shown in most studies to be proapoptotic, inducing apoptosis in immune cells by targeting mitochondria [8, 24–26]. Basic amphipathic helix present in the C-terminal region of PB1-F2 protein dissipates mitochondrial inner membrane potential and brings about apoptosis [27]. Regardless of these findings, the precise mechanism and function of PB1-F2 induced apoptosis and innate immune responses remain unclear. Most studies on PB1-F2 protein are with well-known laboratory strains of influenza viruses, thus, causing lack in knowledge on wide spectrum of IAV subtypes [24, 25]. Hence, to improve our understanding, we analyzed the amino acid sequences of PB1-F2 protein from various IAVs and compared their ability to induce apoptosis and inflammation. In this study, we have used a cell line derived from human lung epithelial cells as an* in vitro *model to study the host responses of influenza PB1-F2 proteins. This is because lung epithelial cells are the key target of influenza virus. Virions initially replicate in airway epithelial cells, which are later released from their apical side towards airspace where they encounter immune cells [28].

Reasons for IAVs to induce apoptosis have been debatable [29–31]. Apoptosis is a multifactorial event and in this study our first objective was to understand the role of PB1-F2 in inducing apoptosis and identify the pathway involved. We found that PB1-F2 protein was minimally apoptotic in A549 cells. This is in congruence with previous studies, where PB1-F2 caused minimal apoptosis in epithelial cells [6, 24]. PB1-F2 constructs from H1N1, H9N2, and H11N1 viruses were more apoptotic than the two highly pathogenic H5N1 strains. Expression analysis of genes gave evidence that PB1-F2 protein brought about apoptosis of epithelial cells via death receptor signaling pathway. Among the death receptor ligands, expression of* TNFα *and* TRAIL* (*TNFSF10*) was significantly upregulated for the PB1-F2 constructs from H1N1, H9N2, and H11N1 (Figure 4). Expression of* FasL* was significantly upregulated in cells transfected with PB1-F2 construct from H9N2 subtype (Figure 4). Although death receptor ligands were significantly upregulated after transfection, there was feeble upregulation of Caspase 8 in H9N2 (Figure 5). This could be because TNF*α* and other death receptor ligands were induced during late stage of transfection. However, Caspase 8 showed statistically significant fold increase in cells transfected with H1N1 and H11N1 PB1-F2 constructs when compared to the mock (empty vector) control (Figure 5).

There was no apparent change in the levels of cytochrome c (data not shown) when measured by Human Cytochrome c Platinum ELISA kit (eBioscience; Affymetrix Co., USA). In contrast to the above, it has been hypothesized earlier that PB1-F2 along with apoptotic stimuli like TNF*α* sensitizes the cells to apoptosis via the proapoptotic effect of BID [24]. Cross-talk between the death receptor-mediated pathway and mitochondrial apoptotic pathway occurs when active Caspase 8 cleaves BID and the truncated BID translocates to the mitochondria causing release of cytochrome *c* and hence apoptosis [31]. Interestingly, there was 20 fold increase in the expression of BIRC3/cIAP2 protein in cells transfected with H5N1-WB PB1-F2 construct (Figure 5(b)). BIRC3 is a member of the IAP family of proteins that inhibits apoptosis by binding to tumor necrosis factor receptor-associated factors TRAF1 and TRAF2, probably by interfering with activation of ICE-like proteases [32]. This could explain to some extent the least amount of apoptosis shown by A549 cells transfected with PB1-F2 from H5N1 subtype. We hypothesize that probably mitochondria are not permeabilized by PB1-F2 protein and the intrinsic pathway is not activated via BID in A549 cells. The minimal apoptosis brought about by PB1-F2 protein is via the extrinsic pathway wherein the Caspase 8 directly activates the executioner caspases 3, 6, and 7. The absence of amino acids essential for mitochondrial localization (L61, L75) in H5N1-PB1-F2 further explains its inability to induce apoptosis through intrinsic pathway (Figure 1).

The second objective of the study was to unravel the strain specific inflammatory response against PB1-F2 proteins. We observed that only H5N1-WB with complete PB1-F2 sequence showed a distinct pattern of cytokine/chemokine induction. H5N1-WB was the strongest inducer of five chemokines/cytokines: CXCL10, CCL5, CCL2, IFN*α*, and IL-6 (Figure 7). The mechanism that promotes high proinflammatory response in H5N1 influenza virus infection is not completely understood, but it is thought to be caused by specific amino acids in the PB1-F2 gene which facilitate this dysregulation of innate immune response [10, 14]. Presence of motifs L62, R75, R79, and L82 in the C-terminal of PB1-F2 gene in H3N2 viruses caused significant pathogenicity in mice [23, 33]. PB1-F2 of H5N1-WB carries this inflammatory motif, hence reiterating the significance of motif. However, it was worth noting that PB1-F2 of the other H5N1-M strain with a natural truncation in N-terminal end, carrying the inflammatory motif in the C-terminal end did not incite heightened levels of chemokines/cytokines [17, 18]. This highlighted the fact that N-terminal end of PB1-F2 has definite role in induction of inflammatory response and it needs to be explored in another study.

Early innate antiviral immune response primarily relies on the production of type I IFN [8]. PB1-F2 has been reported to modulate the induction of IFN in cell type or virus isolate specific manner. There are divergent findings suggesting ambiguities about the antagonistic or agonistic role of PB1-F2 in regulating the IFN response. One study, suggested PB1-F2 mediated exacerbation of IFN-*β* expression in human lung epithelial cells, while another study demonstrated prevention of transcriptional upregulation of type I IFN and other interferon stimulating genes which was associated with interaction of C-terminal domain of PB1-F2 with the mitochondrial antiviral signaling protein (MAVS) [8]. In our study, PB1-F2 construct from H5N1-WB strain caused upregulation of IFN*α* and the levels were significantly higher than the cell and mock control. In contrast, PB1-F2 construct from H1N1-WSN strain prevented induction of IFN*α* in A549 cells. Thus, our result also proves that PB1-F2 brings about strain specific regulation of type1 IFN (Figure 7). It is very interesting to note that PB1-F2 construct from H5N1-WB initially exacerbated the IFN*α* levels which is detrimental to successful virus infection and then induced aberrant chemokine production thus supporting the hypothesis that H5N1 pathogenicity arises from the fatal effects of hypercytokinemia. We believe that PB1-F2 contributes significantly to this high pathogenicity. This is supported by our previous study, where we observed that approximately 96% of the H5N1 strains possessed complete PB1-F2 fragment. This suggests that PB1-F2 is positively selected in these strains and definitely necessary for the virus survival and proliferation [9].* In silico* prediction of the secondary structure of the PB1-F2 from the strains used in this study revealed substantial differences in the secondary structure of the C-terminal regions of PB1-F2 proteins. We speculate that these differences in the conformations will definitely have bearing on the varying inflammatory response displayed by the proteins. However, these observations need to be confirmed by more sophisticated technology like NMR and CD spectroscopy.

Thus, our study suggests that ectopic expression of PB1-F2 induces minimal apoptosis in A549 lung epithelial cells. The induction of apoptosis is through death receptor mediated extrinsic pathway. PB1-F2 protein from H5N1 subtype induced least apoptosis but maximum proinflammatory response. This study emphasizes the strain specific agonist or antagonist effect of PB1-F2 on the expression of IFN*α* in A549 cells. Apart from C-terminal region of PB1-F2, N-terminal portion is also essential to induce exuberant proinflammatory response. This study was carried out using PB1-F2 constructs and not using any wild type or recombinant viruses created by reverse genetics. We believe this is advantageous, mainly because of the overlapping nature of the viral ORFs and the interdependence in expression of PB1, PB1-F2, and PB1-N40, which makes it difficult to evaluate the contribution of individual protein [34]. This study is an attempt to fill in the gap in the literature regarding the effect of PB1-F2 from various IAV strains.

## Figures and Tables

**Table 1:** Primer sequences used in the study.

**Primer**	**Sequences 5' to 3'**	**Size (bp)**
Uni12	AGCRAAAGCAGG	

H1N1	F- CTTACAGCCATGGGACAGGAACAGG	270 bp
	R- CTTGTGTAAGCTTGTCCACTCGTGT	

H5N1	F- ACAGCCATGGAACAGGGACAGGATACA	270bp
	R- GACCTTGGGTGAGTTTATCCACTCTT	

H5N1-F2	F- ACCCAATTGATGGACCATTACCTGAG	156bp
	R- GACCTTGGGTGAGTTTATCCACTCTT	

H11N1	F- CATACAGCCATGGAACAGGAACAGG	270bp
	R- CTTGGGTGAGTTTCTTGGGTGAGTTTGTCCACTCTTGT	

## Data Availability

The data used to support the findings of this study are included within the article.
